# Data from expressed sequence tags from the organs and embryos of parthenogenetic *Haemaphysalis longicornis*

**DOI:** 10.1186/s13104-021-05740-3

**Published:** 2021-08-25

**Authors:** Rika Umemiya-Shirafuji, Jinlin Zhou, Min Liao, Badgar Battsetseg, Damdinsuren Boldbaatar, Takeshi Hatta, Thasaneeya Kuboki, Takeshi Sakaguchi, Huey Shy Chee, Takeharu Miyoshi, Xiaohong Huang, Naotoshi Tsuji, Xuenan Xuan, Kozo Fujisaki

**Affiliations:** 1 National Research Center for Protozoan Diseases, Obihiro University of Agriculture and Veterinary Medicine, Inada-Cho, Obihiro, Hokkaido 080-8555 Japan; 2 Shanghai Veterinary Research Institute, Chinese Academy of Agricultural Sciences, No.518, Ziyue Road, Minhang District, Shanghai, 200241 China; 3 Laboratory of Molecular Genetics, Institute of Veterinary Medicine, Mongolian University of Life Science, Zaisan, 17024 Ulaanbaatar, Mongolia; 4 Institute of Veterinary Medicine, Mongolian University of Life Science, Zaisan, 17024 Ulaanbaatar, Mongolia; 5 Laboratory of Parasitic Diseases, National Institute of Animal Health, National Agricultural and Food Research Organization, 3-1-5 Kannondai, Tsukuba, Ibaraki 305-0856 Japan; 6 Department of Parasitology and Tropical Medicine, Kitasato University School of Medicine, 1-15-1 Kitasato, Minami, Sagamihara, Kanagawa 252-0374 Japan; 7 Institute for Materials Chemistry and Engineering, Kyushu University, 744 Motooka, Nishi-ku, Fukuoka, 819-0395 Japan

**Keywords:** Tick, *Haemaphysalis longicornis*, Embryo, Fat body, Hemolymph, Midgut, Ovary, Salivary glands, Expressed sequence tags, Full-length cDNA library

## Abstract

**Objectives:**

*Haemaphysalis longicornis* is the most important tick species in Japan and has a wide range of vector capacity. Due to its veterinary and medical importance, this tick species has been used as a model for tick/vector biological studies. To identify the key molecules associated with physiological processes during blood feeding and embryogenesis, full-length cDNA libraries were constructed using the fat body, hemocytes-containing hemolymph, midgut, ovary and salivary glands of fed females and embryos of the laboratory colony of parthenogenetic *H. longicornis*. The sequences of cDNA from the salivary glands had been already released. However, the related information is still poor, and the other expressed sequence tags have not yet been deposited.

**Data description:**

A total of 39,113 expressed sequence tags were obtained and deposited at the DNA DataBank of Japan. There were 7745 sequences from embryos, 7385 from the fat body, 8303 from the hemolymph including hemocytes, 7385 from the midgut, and 8295 from the ovary. The data, including expressed sequence tags from the salivary glands was summarized into Microsoft Excel files. Sharing this data resource with the tick research community will be valuable for the identification of novel genes and advance the progress of tick research.

## Objective

*Haemaphysalis longicornis* is an important tick species, and is a vector for various pathogens affecting humans and animals in Asia and Oceania. In the veterinary field, the tick species is a major pest of cattle, because it can spread *Theileria orientalis*, a protozoan parasite, which causes piroplasmosis and produces economic losses to livestock industry producers. There are no anti-tick vaccines or therapeutic agents against *T. orientalis* infection available at present in Japan. *H. longicornis* also occurs in Australia, New Zealand, New Caledonia, the Fiji Islands, Korea, China and Russia [1]. Although the tick species was not detected outside of quarantine until 2017, a heavy infestation of *H. longicornis* was recently reported in the United States [2]. *H. longicornis* is a vector of not only bovine piroplasmosis, but also canine babesiosis caused by *Babesia* parasites, and rickettsiosis and viral diseases in humans. Throughout its distribution, *H. longicornis* is an increasing threat to livestock animals and humans.

*Haemaphysalis longicornis* has been used as a model for tick/vector studies. As a development platform for novel control strategies, including anti-tick vaccines, bio-acaricides and anti-protozoal drugs against ticks and tick-borne diseases, we have constructed full-length cDNA libraries using laboratory-reared parthenogenetic *H. longicornis*. The expressed sequence tags (ESTs) in these libraries have made it possible to identify cDNA sequences which may be used to elucidate molecular processes such as blood digestion, oxidative stress, apoptosis, reproduction, and survival [3–8]. Currently, only 8471 EST sequences of the salivary glands are available in public databases [9]; information regarding the other sequences has not been shared yet. This situation means that the extension and improvement of tick research is limited. The data should be shared worldwide, because of its veterinary and medical importance.

## Data description

The full-length cDNA library was made using the vector-capping method [11]. The construction of each cDNA library has previously been reported [4, 5]. The parthenogenetic tick *H. longicornis* (Okayama strain) was maintained at the National Research Center for Protozoan Diseases, Obihiro University of Agriculture and Veterinary Medicine, and was fed on the ears of Japanese white rabbits (Japan SLC, Shizuoka, Japan) using the cotton bag method [10]. Female ticks which had been fed from three to four days (corresponding to the rapid feeding stage) were dissected in cold phosphate-buffered saline (137 mM NaCl, 2.7 mM KCl, 10 mM Na_2_HPO_4_, 1.8 mM KH_2_PO_4_, pH 7.4), and the fat body, hemolymph including hemocytes, midgut, and salivary glands were pooled for each organ. The ovary samples were collected from both partially-engorged (four to five-day-fed) and engorged female ticks, and pooled. Eggs laid at the third to fourth day after the onset of oviposition were collected and incubated at 28 °C for seven to eight days to develop. The samples were homogenized using a pestle in TRI reagent (Sigma-Aldrich, MO, USA). Total RNA extraction was performed using TRI reagent, according to the manufacturer’s protocol. cDNA was synthesized from 5 μg of total RNA using the G-Capping method [11], and ligated into the plasmid vector pGCAP1. The resulting plasmids were transformed into *Escherichia coli* DH12S (Thermo Fisher Scientific, MA, USA). A total of 10,000 recombinant transformants from the library were randomly selected for plasmid DNA purification and sequencing. The nucleotide sequences were determined using an automated sequencer (ABI PRISM 310 Genetic Analyzer; Thermo Fisher Scientific) and then analyzed for identity using the BLASTX program (National Center for Biotechnology Information (NCBI); https://blast.ncbi.nlm.nih.gov/Blast.cgi). The ESTs were constructed by random partial sequencing of the 5’-terminal of the cDNA clones from each cDNA library.

A total of 39,113 ESTs obtained were deposited in the DNA DataBank of Japan (DDBJ) [12]. The deposited sequences contained 7745 ESTs from embryos (Table 1, Data file 12) [13], 7385 from the fat body (Table 1, Data file 7) [14], 8303 from the hemolymph including hemocytes (Table 1, Data file 8) [15], 7385 from the midgut (Table 1, Data file 9) [16], and 8295 from the ovary (Table 1, Data file 10) [17]. Sample information was deposited in the DDBJ BioSample database (Table 1, Data files 7–10 and 12) [13–17]. The results of a homology search of EST sequences using the BLASTX program were summarized and input into an MS Excel file for each organ (Table 1, Data files 1–4 and 6) [18–21, 23]. For salivary glands, the descriptions of the BLASTX search results for 6,347 of 8,471 sequences previously released are listed in data files 5 and 11 (Table 1) [9, 22]. The Excel files contain accession numbers, entry names, and the BLASTX search results, which are also downloadable on our website (https://www.obihiro.ac.jp/facility/protozoa/en/project/project-ticks).

**Table 1:** Overview of data files/data sets

Label	Name of data file/data set	File types (file extension)	Data repository and identifier (DOI or accession number)
Data file 1	ESTs_Hl_Fat Body	MS Excel file (.xlsx)	Obihiro University Archives of Knowledge (http://doi.org/10.24556/00004700) [18]
Data file 2	ESTs_Hl_Hemolymph	MS Excel file (.xlsx)	Obihiro University Archives of Knowledge (http://doi.org/10.24556/00004701) [19]
Data file 3	ESTs_Hl_Midgut	MS Excel file (.xlsx)	Obihiro University Archives of Knowledge (http://doi.org/10.24556/00004702) [20]
Data file 4	ESTs_Hl_Ovary	MS Excel file (.xlsx)	Obihiro University Archives of Knowledge (http://doi.org/10.24556/00004703) [21]
Data file 5	ESTs_Hl_Salivary glands	MS Excel file (.xlsx)	Obihiro University Archives of Knowledge (http://doi.org/10.24556/00004704) [22]
Data file 6	ESTs_Hl_Embryo	MS Excel file (.xlsx)	Obihiro University Archives of Knowledge (http://doi.org/10.24556/00004705) [23]
Data file 7	Hl FB full-length cDNA library	FASTA	DDBJ/ENA/GenBank (Accession numbers: HY961648-HY969032) https://identifiers.org/ncbi/insdc:HY961648 [14]
Data file 8	Hl HE full-length cDNA library	FASTA	DDBJ/ENA/GenBank (Accession numbers: HY969033-HY977335) https://identifiers.org/ncbi/insdc:HY969033 [15]
Data file 9	Hl MG full-length cDNA library	FASTA	DDBJ/ENA/GenBank (Accession numbers: HY977336-HY984720) https://identifiers.org/ncbi/insdc:HY977336 [16]
Data file 10	Hl OV full-length cDNA library	FASTA	DDBJ/ENA/GenBank (Accession numbers: HY984721-HY993015) https://identifiers.org/ncbi/insdc:HY984721 [17]
Data file 11	Hl Sg full-length cDNA library	FASTA	DDBJ/ENA/GenBank (Accession numbers: DC574924-DC583394) https://identifiers.org/ncbi/insdc:DC574924 [9]
Data file 12	Hl EM full-length cDNA library	FASTA	DDBJ/ENA/GenBank (Accession numbers: HY953903-HY961647) https://identifiers.org/ncbi/insdc:HY953903 [13]

## Limitations

- Total RNA was extracted from each organ of three to four-day fed (corresponding to the rapid feeding stage) or partially-engorged and engorged female ticks of parthenogenetic *H. longicornis*. The ESTs were determined based on full-length cDNA libraries from organs, and their data files are useful in the search for novel homologous genes expressed at the rapid feeding and engorgement periods. While the data in this study are informative, they cannot be used for comparisons with data derived from others, such as samples from the unfed periods or bisexual population.
- Multi-omics data, which are valuable, powerful tools for tick research, are still limited for *H. longicornis* ticks, leading to a delay in cutting-edge research, compared to research carried out on *Ixodes scapularis* and *Rhipicephalus* (*Boophilus*) *microplus* ticks. Recently, a New Zealand-USA consortium was established to sequence, assemble, and annotate the genome of *H. longicornis* ticks obtained from New Zealand's North Island [24]. The genomic data of *H. longicornis* ticks from China was released [25]. Due to current unavailability of their annotation information, we updated the annotation for each EST database using the BLASTX program in the present study. Because *H. longicornis* is unique among ticks, having both triploid parthenogenetic and diploid bisexual races, continuous obtaining of related-data will be required for characterizing this species. The ESTs of our laboratory strain of parthenogenetic *H. longicornis* will facilitate a better understanding of the biology and physiology of this tick species.

## Data Availability

The data described in this Data Note can be freely and openly accessed at the DNA DataBank of Japan (DDBJ) under DC574924-DC583394 and HY953903-HY993015, which are shared by DDBJ/ENA/GenBank [9, 13–17]. Please see Table 1 and references [9, 13–17] for details and links to the data. EST sequences (accession numbers DC574924-DC583394) of the fat body, hemolymph including hemocytes, midgut, ovary and embryos have been deposited in BioProject https://www.ncbi.nlm.nih.gov/bioproject/705904. The Microsoft Excel data files generated in the current study are available at the Obihiro University Archives of Knowledge (OAK), an open access repository at the Obihiro University of Agriculture and Veterinary Medicine (https://obihiro.repo.nii.ac.jp/): Data file 1_ESTs_Hl_Fat Body (http://doi.org/10.24556/00004700) [18], Data file 2_ESTs_Hl_Hemolymph (http://doi.org/10.24556/00004701) [19], Data file 3_ESTs_Hl_Midgut (http://doi.org/10.24556/00004702) [20], Data file 4_ESTs_Hl_Ovary (http://doi.org/10.24556/00004703) [21], Data file 5_ESTs_Hl_Salivary glands (http://doi.org/10.24556/00004704) [22], Data file 6_ESTs_Hl_Embryo (http://doi.org/10.24556/00004705) [23].

## Abbreviations

BLAST: Basic Local Alignment Search Tool
DDBJ: DNA DataBank of Japan
ENA: European Nucleotide Archive
EST: Expressed sequence tag
NCBI: National Center for Biotechnology Information
